# Self-Filtering Monochromatic Infrared Detectors Based on Bi_2_Se_3_ (Sb_2_Te_3_)/Silicon Heterojunctions

**DOI:** 10.3390/nano9121771

**Published:** 2019-12-12

**Authors:** Xujie Pan, Jing He, Lei Gao, Handong Li

**Affiliations:** School of Materials and Energy, University of Electronic Science and Technology of China, Chengdu 610054, China

**Keywords:** Bi_2_Se_3_, Sb_2_Te_3_, Si photovoltaic detector, external quantum efficiency, response time

## Abstract

This paper focuses on the photoelectric properties of heterostructures formed by surface-modified Si (111) and hexagonal, quintuple-layered selenides (Bi_2_Se_3_ and Sb_2_Te_3_). It was shown that H-passivated Si (111) can form robust Schottky junctions with either Bi_2_Se_3_ or Sb_2_Te_3_. When back illuminated (i.e., light incident towards the Si side of the junction), both the Bi_2_Se_3_/Si and Sb_2_Te_3_/Si junctions exhibited significant photovoltaic response at 1030 nm, which is right within the near-infrared (NIR) light wavelength range. A maximum external quantum efficiency of 14.7% with a detection response time of 2 ms for Bi_2_Se_3_/Si junction, and of 15.5% with a 0.8 ms response time for the Sb_2_Te_3_/Si junction, were achieved. Therefore, utilizing Si constituents as high-pass filters, the Bi_2_Se_3_ (Sb_2_Te_3_)/Si heterojunctions can serve as monochromatic NIR photodetectors.

## 1. Introduction

Topological insulators (TIs) possess helical surface states locked by time-reversal symmetry (TRS), and have been predicted to be promising candidates for electronic and optoelectronic device applications [1,2,3,4]. Moreover, the growth of these TIs on Si substrates has been optimized, and such heterostructures were shown to exhibit a Schottky junction which has already been widely studied [5,6,7]. However, most research has focused on the growth and optoelectronic properties of TIs. In fact, combining TI with other materials is also worthy of attention. Silicon is well known for its application in near-infrared optical detection due to its low dark current fast response and easy integration. Near-infrared light detectors are typically based on diode or Schottky structures; the key unit of a diode detector is its P–N or PIN junction, which requires a basic bias voltage. Although the diode detector has a large dark current and low response speed, its quantum efficiency is high. Compared to those simple diode-based detectors, Schottky junction-based detectors require metal to couple with silicon; compared with PIN detection, the latter has a faster response speed, but also has the problem of a low potential barrier, which causes a huge leakage of current. In addition, the problem of reactions between metal and silicon at the interface cannot be ignored, as it may cause poor device performance. Based on these issues, we innovatively propose the use of Bi_2_Se_3_ and Sb_2_Te_3_ to construct a heterostructure with Si. The layered nature of these two materials can offer a good interfacial characteristic; the performance of the Schottky junction is also likely to be further improved. On the other hand, the band gap of Si lies in the near infrared wavelength region (~1.12 eV), which implies that photons in the infrared region can be absorbed by Si in principle. However, for practical applications, monochromatic optical detectors are more desirable. So, some more work is still to be done. The traditional method is to add a filter structure to filter out the light of high frequencies, but this increases the difficulty of implementing the device. Meanwhile, the filter or membrane structure is costly, and therefore, not conducive to commercialization. As an indirect bandgap semiconductor, the absorption coefficient of Si for different frequencies photons varies greatly. For example, light with a 600 nm wavelength cannot penetrate a Si substrate with a thickness of 300 microns, while a wavelength of 1000 nm can still have 25% strength after passing through the Si substrate of the same thickness. Therefore, selective high-pass filtering can be realized by adjusting the thickness of the Si substrate. In this report, the photoelectric effect of the heterostructure formed by Bi_2_Se_3_ and Sb_2_Te_3_ with P–Si (111) is analyzed. The high wavelength light was incident from the Si substrate side, which is transparent to wavelengths. The substrate of Si as a high-pass filter formed a near infrared monochromatic light detector with the Bi_2_Se_3_ and Sb_2_Te_3_ film. The external quantum efficiency and response speed of the photodetector are also analyzed. These results demonstrated the great potential of this TI/Si heterostructure for high-performance optoelectronic device applications.

## 2. Materials and Methods

The high quality, single-crystalline Bi_2_Se_3_ thin film on a Si (111) substrate was grown by molecular beam epitaxy (MBE), whereas the Sb_2_Te_3_ film was grown using the physical vapor deposition (PVD) technique [8,9]. As a large lattice mismatch exists between Bi_2_Se_3_ and Si (111), surface modification engineering was employed for Si (111). H-passivated Si (111) was obtained by rinsing Si (111) in hydrofluoric acid (HF) for 10 min after a standard cleaning procedure. This H-passivated Si (111) was then loaded into a MBE chamber and degassed for 24 h to get rid of the impurities and obtain a clean surface. The surface structure of Si (111) was modified by flashing at high temperature (over 1000 °C with Si exhibiting a bright orange-yellow color) for about 30 s. Bi was grown firstly to further minimize the lattice mismatch, followed by the growth of the Bi_2_Se_3_ film. Sb_2_Te_3_ film was prepared on a H-passivated Si (111) substrate via PVD. The Bi_2_Se_3_ film was prepared on Bi-passivated Si (111). After the growth, the crystalline quality was studied by High resolution X-ray diffraction (XRD, Bede 1, from Jordan Valley Semiconductors). The photodetector device from the Bi_2_Se_3_ on H-passivated Si (111) (BS/H–Si) was fabricated to analyze its photoelectric properties. A self-filtered silicon Schottky monochrome detector was fabricated according to the following steps. The device has four layers. The first layer is a window electrode. The window electrode comprises a Ga-In alloy to make ohmic contact with the Si substrate. The second layer is the Si substrate with a low-pass filter function. The Si substrate was single crystalline along (111) orientation, with both sides polished, a thickness of 300 μm, and a resistance of 1~10 Ω/cm. The third layer consisted of a metal film (typically Bi_2_Se_3_ or Sb_2_Te_3_) with high infrared reflectivity. Furthermore, the metal film here is Bi_2_Se_3_ or Sb_2_Te_3_ film grown along a (001) crystalline direction, with a thickness ranging from 50 nm to 200 nm. Indium was used as the bottom electrode to form ohmic contact with the Bi_2_Se_3_ or Sb_2_Te_3_ film. An external bias was applied on the sample device using source meter (Keithley 2400, from Tektronix, Beaverton, Oregon, USA). Then, solar light from a class 3A sun simulator was used to shine light vertically incident on the sample from the Si substrate side. Bi_2_Se_3_ on the Bi-passivated Si (111) (BS/Bi–Si), Sb_2_Te_3_ H-passivated Si (111) device was fabricated and tested in the same way. QEX 10 equipment was employed to study the photoelectric effect. The surface morphology of the samples grown by MEB was observed by in situ scanning tunneling microscopy (STM).

## 3. Results

**Figure 1:**
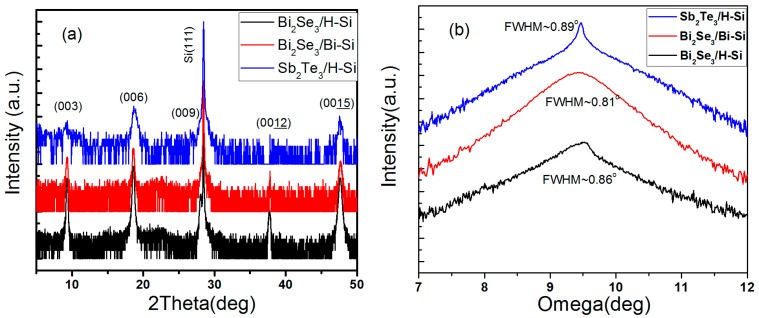
XRD spectrum of Bi_2_Se_3_ and Sb_2_Te_3_ thin film on a Si (111) substrate. (**a**) 2Theta scan, and (**b**) rocking curve of Bi_2_Se_3_ (006) and Sb_2_Te_3_ (006). The blue line represents Sb_2_Te_3_ film on H–Silicon, the red line represents Bi_2_Se_3_ film on Bi–Silicon; the black line represents the Bi_2_Se_3_ film on H–Silicon.

Bi_2_Se_3_ and Sb_2_Te_3_ were grown on H-passivated Si (111) via MBE and PVD respectively. During the growth of Bi_2_Se_3_ via MBE, an additional Bi layer was grown on H-passivated Si (111) as a buffer layer to minimize the lattice mismatch. The crystal structures of the as-grown films were studied by XRD. The XRD patterns are displayed in Figure 1a; they demonstrate diffraction peaks corresponding to the (00*l*) planes of the Bi_2_Se_3_ and Sb_2_Te_3_. The XRD results indicate that the Bi_2_Se_3_ and Sb_2_Te_3_ films are crystalline, and of high quality, as demonstrated by the full width at half maxima (FWHMs) of the (006) plane of the Bi_2_Se_3_ and Sb_2_Te_3_ film.

**Figure 2:**
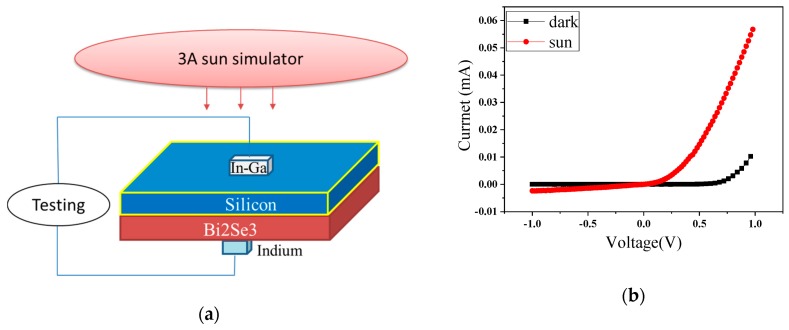

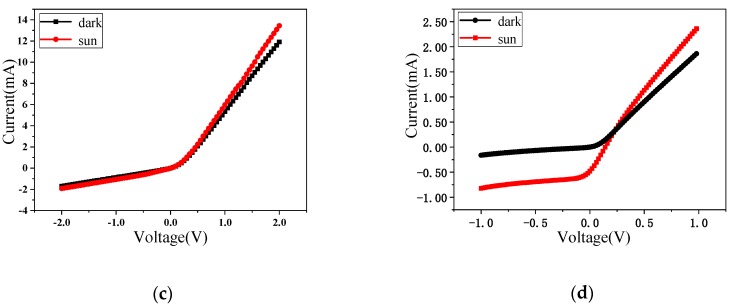
(**a**) Schematic of BS/Si heterostructure photodetector (**b**) I-V curve of BS/H–Si heterojunction (**c**) I-V curve of BS/Bi–Si heterojunction (**d**) I-V curve of ST/H–Si heterojunction in the under illumination and dark.

Figure 2a presents a schematic illustration of the Bi_2_Se_3_/H–Si device under illumination. Figure 2b shows the current-voltage (I-V) characteristics of the device under sunlit and dark conditions at room temperature while and external bias voltage is applied. According to the typical diode characteristics, it can be concluded that the film formed a robust Schottky contact with the P–Silicon substrate. The curve is also in good accordance with Richardson-Dushman thermionic emission theory [10,11,12]. It is worth noting that the light was incident from the Si substrate side, in order to study the Bi_2_Se_3_/H–Si heterostructure. This is because the strong reflection and absorption of Si in the visible range can be used as a natural low-pass filter for the device [13]. Part of the light passed through the Si substrate and reached the heterojunction, and the different type of junctions caused the I-V curve to change. Furthermore, an evident photoelectric effect was observed in the Bi_2_Se_3_/H–Si heterostructure, from which we can note that the open circuit voltage (Voc) was 0.16 V, the short circuit current (Isc) was 0.05 mA, and the photoelectric conversion efficiency was 0.083%.

**Figure 3:**
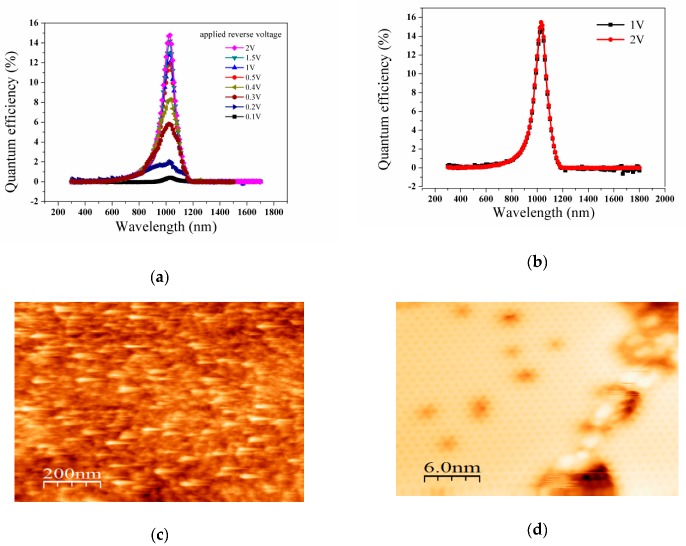
(**a**) The EQE of BS/H–Si heterostructure (**b**) The EQE of ST/H–Si heterostructure (**c**) STM of H–Si (111) (**d**) STM of Bi–Si (111).

Figure 2c shows the typical current-voltage curve, from which we can determine that a Schottky contact exists between BS and Bi–Si. However, when light was incident from the substrate, the current changed slightly. For the heterostructure formed by the Sb_2_Te_3_ film with H-passivation Si (ST/H–Si), the same test was done. Figure 2d indicates that ST/H–Si formed a Schottky contact and had a photoelectric conversion efficiency when the solar was incident from the substrate side. Figure 3a shows that the quantum efficiency of the BS/H–Si heterojunction changed significantly at wavelengths between 800 nm and 1200 nm. However, at other wavelengths, the external quantum efficiency was not even detected. This indicates that when the wavelength of light was larger than 1200 nm, electrons could not be excited, but at less than 800 nm, photons could not reach the heterojunction due to being blocked by the silicon. The effect of different reverse bias voltages applied on the BS/H–Si heterojunction were also studied. When the reverse voltage was 0.1 V, the external quantum efficiency was low. But when it increased to 0.5 V, it increased rapidly, indicating that the space charge layer had enlarged. However, as the voltage continued to increase, the external quantum efficiency increased slowly. Furthermore, the sample broke down when the reverse voltage was more than 2 V. This indicated that the space charge layer was nearly saturated at 2 V. Meanwhile, the maximum value reached 14.7% when the wavelength was 1030 nm and the reverse voltage was 2 V. The ST/H–Si heterojunction also had a similar external quantum efficiency as that shown in Figure 3b. Obvious quantum efficiency occurred between 800 nm and 1200 nm, when the reverse voltage was applied. The space charge layer was approaching saturation at 2 V. Meanwhile, the quantum conversion efficiency was the highest at 1030 nm, reaching 15.5%, under the same reverse voltage.

STM can provide us with surface morphology information. From the Figure 3c, the surface of H-passivated Si (111) is atomic rough, which indicates that monohydride, dihydride, and trihydride coexist on the surface of H-passivated Si [14]. The H–Si bonding causes the first silicon layer to be slightly relaxed inward [15]. This indicates that the lattice constant of Si becomes larger when H bonds with Si, resulting in the narrowing of the band gap. The band gap of silicon at room temperature was 1.12 eV, and light longer than 1100 nm cannot be absorbed [16]. However, due to the compression of the band gap, light with a wavelength of 1100 nm to 1200 nm can be absorbed. The H-passivated surface of Si near the film was protected, but the other side which was exposed to the air was oxidized [17]; this led to the lattice constant having no change. Silicon with different surface lattice constants is formed, which results in external quantum efficiency at 1100 nm to 1200 nm. Light from 800 nm to 1000 nm has low transmittance and high absorptance when passing through the Si substrate, while light from 1000 nm to 1200 nm has high transmittance and low absorptance. Considering these two effects, it shows that an external quantum efficiency peak appears at 1030 nm. The Bi_2_Se_3_ film has a good reflectivity in the near-infrared waveband, and the light passing through the substrate is reflected back to the substrate by the film, causing secondary absorption, which increases the external quantum efficiency value, but causes the external quantum efficiency peak to be incompletely symmetric.

**Figure 4:**
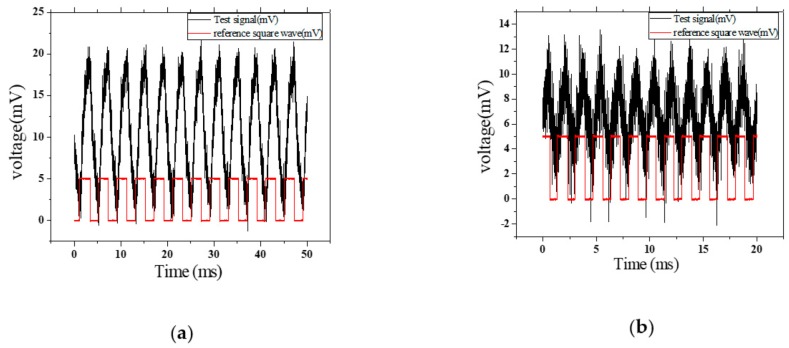
(**a**) Response speed of BS/H–Si under 250 Hz light pulse, and (**b**) response speed of ST/H–Si under 600 Hz light pulse.

From Figure 4a, the BS/H–Si device exhibited a fast response at 250 Hz pulse. So, the response speed in one cycle was studied under a 250 Hz light pulse; the rising time and decay time were 2 ms. The response speed of the ST/H–Si device was tested. As shown in Figure 4b, the response speed in one cycle was studied under a 600 Hz light pulse; the rising time and decay time was 0.8 ms.

## 4. Discussion

To further explain the photocurrent generation process, the Bi_2_Se_3_/H–Si heterostructure was selected as an example. The presence of a large number of dangling bonds on the Si (111) surface leads to a high density of surface states [18,19]; however, H-passivation can reduce the density of states on the Si surface effectively [14,15]. Because of the different work function of Si and Bi_2_Se_3_ [20,21], the electrons will flow from the film to the Si, and a space charge layer will be formed at their interface. The incident light stimulates the transition of electrons from the bulk valence band to the empty bulk conduction band or to unoccupied surface-states, which generates electron-hole pairs [22,23]. The electrons and holes are separated by the built-in field at the interface. Electrons were injected into the Bi_2_Se_3_ film and the holes were drifted to the electrodes. Thereby, a photocurrent is generated in the external circuit. The existence of the built-in electric field at the interface reduces the recombination of hole carriers, which extends the lifetime of the carriers. The increasing reverse voltage causes the space charge layer to expand, which also promotes external quantum efficiency. The conclusion is consistent with our experimental results. From Figure 3d, the honeycomb structure shows that a Bi trimer was formed on the Si surface which could not reduce the surface state [24]. Because of the high surface state density of Si [18,19], the Fermi level was pinned when Si came into contact with Bi [25], which limited the space charge layer enlargement. The rectification was relatively low, and thus, didn’t show any photoelectric response. This also verifies the phenomena observed in our experiments.

To further explain the photocurrent generation process, the Bi_2_Se_3_/H–Si heterostructure was selected as an example. The presence of a large number of dangling bonds on the Si (111) surface leads to a high density of surface states [18,19]; however, H-passivation can reduce the density of states on the Si surface effectively [14,15]. Because of the different work function of Si and Bi_2_Se_3_ [20,21], the electrons will flow from the film to the Si, and a space charge layer will be formed at their interface. The incident light stimulates the transition of electrons from the bulk valence band to the empty bulk conduction band or to unoccupied surface-states, which generates electron-hole pairs [22,23]. The electrons and holes are separated by the built-in field at the interface. Electrons were injected into the Bi_2_Se_3_ film and the holes were drifted to the electrodes. Thereby, a photocurrent is generated in the external circuit. The existence of the built-in electric field at the interface reduces the recombination of hole carriers, which extends the lifetime of the carriers. The increasing reverse voltage causes the space charge layer to expand, which also promotes external quantum efficiency. The conclusion is consistent with our experimental results. From Figure 3d, the honeycomb structure shows that a Bi trimer was formed on the Si surface which could not reduce the surface state [24]. Because of the high surface state density of Si [18,19], the Fermi level was pinned when Si came into contact with Bi [25], which limited the space charge layer enlargement. The rectification was relatively low, and thus, didn’t show any photoelectric response. This also verifies the phenomena observed in our experiments.

## 5. Conclusions

In summary, single-crystalline Bi_2_Se_3_ and Sb_2_Te_3_ were grown on a H-passivated P–Si (111) successfully, and the film quality was analyzed by XRD. The heterostructure of the Bi_2_Se_3_/H–Si and Sb_2_Te_3_/H–Si photodetectors showed an obviously photoelectric effect when the light was incident from the substrate side. Meanwhile, the heterostructure of Bi_2_Se_3_/H–Si and Sb_2_Te_3_/H–Si possessed high quantum efficiency between 800 nm and 1200 nm and obvious response speeds. This indicated that photodetectors based on Bi_2_Se_3_/H–Si and Sb_2_Te_3_/H–Si have bright prospects in the future.
